# Is access to euthanasia drugs and moral stress linked to suicide rates in veterinarians? A cross-sectional national survey and network analysis

**DOI:** 10.3389/fpsyt.2026.1795845

**Published:** 2026-05-20

**Authors:** J. Rymaszewska, K. Fila-Pawłowska, D. Szcześniak, W. Hildebrand, E. Pawłowska, M. Magdziarz

**Affiliations:** 1 Department of Clinical Neurosciences, Wroclaw University of Science and Technology, Wroclaw, Poland; 2 Department of Psychiatry, Wroclaw Medical University, Wroclaw, Poland; 3 National Veterinary Medical Council, Warsaw, Poland; 4 Student Association, Medical Department, University of Zielona Góra, Zielona Góra, Poland; 5 Hugo Steinhaus Center, Faculty of Pure and Applied Mathematics, Wroclaw University of Science and Technology, Wroclaw, Poland

**Keywords:** acquired capability for suicide, euthanasia, moral stress, suicide risk, veterinarians

## Abstract

**Introduction:**

Veterinarians face unique occupational risks contributing to elevated suicide rates, including psychological distress, moral stress from euthanasia, and access to lethal drugs. No national study has simultaneously modelled the combined contributions of lethal means access, moral stress, and psychopathology to suicide risk in this population using machine learning approaches.

**Methods:**

This national cross-sectional study of 783 Polish veterinarians used validated psychometric tools and machine learning to identify predictors of suicide risk.

**Results:**

Nearly 42% exceeded the clinical cut-off for suicide risk, and 23.7% reported thoughts of using euthanasia drugs on themselves. This variable emerged as the strongest predictor of both suicidal behavior and acquired capability for suicide (β=2.83 for suicidal behavior; β=8.58 for acquired capability, both p<.001, model R²=.467 and .220 respectively), surpassing depression, anxiety, and burnout. Moral stress was associated with age but not directly with suicidality. Network analysis revealed that euthanasia-related ideation formed a critical bridge between suicidal thoughts and behavioral capability.

**Outcomes/discussion:**

Findings highlight the urgent need for profession-specific suicide prevention strategies, including restricted access to euthanasia drugs and targeted mental health interventions. To our knowledge, this is among the first studies to simultaneously model the combined contributions of lethal means access, moral stress, and psychopathology to suicide risk in this population using machine learning approaches.

## 1 Introduction

According to the World Health Organization more than 700,000 people lose their life as a result of suicide every year, suicide being the third leading cause of mortality in 15–29-year-olds globally (1). In Poland, suicide represents a substantial public health burden. According to WHO data, Poland recorded a suicide mortality rate of 23.9 per 100–000 population in 2016 — more than double the global standardized rate of 10.53 per 100,000 for that year — ranking Poland 22nd globally (1, 2). Between 1999 and 2018, over 95,000 people died by suicide in Poland, with a pronounced male predominance (men:women ratio of 5–6:1) and a marked increase from approximately 4000 annual deaths to over 6000 in the years 2013–2015 (2). Despite this burden, profession-specific data on veterinarians in the Polish context remain scarce, representing a notable research gap.

Suicidal thoughts and behaviors (STBs) remain a major public health concern with limited progress in prevention over recent decades (3). A wide range of suicide risk factors has been identified in the literature, however, most are more effective in predicting suicidal ideation than suicidal behavior or suicide death (4). These risk factors span individual, interpersonal, and contextual domains, and that no single factor is sufficient to explain suicidal outcomes. Importantly, as emphasized by the ideation-to-action models (5), the emergence of suicidal thoughts and the progression from ideation to attempt are distinct processes with partly different predictors (6). Common risk factors such as depression, hopelessness, and most mental disorders strongly predict suicidal ideation but are less effective in distinguishing individuals who go on to attempt suicide (6).

The Three-Step Theory (3ST) of suicide, developed by Klonsky and May (7), provides a clear and testable framework for understanding how suicidal ideation progresses to action. According to this model, suicidal ideation emerges when psychological pain is combined with hopelessness. Ideation intensifies when pain exceeds connectedness to life, including relationships, responsibilities, or personal goals. A suicide attempt, however, occurs only when the individual also has the capacity to act, which may arise from dispositional traits (e.g., low fear of pain), acquired experiences (e.g., habituation to self-harm), or practical factors (e.g., access to lethal means).

Other influential frameworks include the Interpersonal Theory of Suicide (IPT), which proposes that suicidal desire emerges from thwarted belongingness and perceived burdensomeness, and becomes more dangerous when accompanied by capability for suicide (8), and the Integrated Motivational-Volitional (IMV) model, which identifies access to means as important volitional moderator in the transition from ideation to attempt (9). The 3ST was selected as the primary framework for the present study because of its explicit inclusion of acquired capability which is directly relevant to occupational familiarity with lethal means, and because its ideation-to-action structure maps closely onto the occupational variables examined here and its relevance to research on veterinarians, a professional group with disproportionately high suicide rates (10, 11). Previous literature indicates that veterinarians frequently report high psychological pain, moral stress, and access to euthanasia drugs (11–13), factors aligned closely with all three steps of the 3ST. Their combination of occupational strain, emotional isolation, and ready access to lethal means may pace this group at particular risk of progressing from suicidal ideation to attempt.

From the perspective of suicide related research, veterinary practitioners constitute a unique population. In some respects, they resemble the general population more than psychiatric samples, yet they are simultaneously exposed to distinctive occupational and environmental risk factors. This combination makes them especially informative for examining the transition from suicidal thoughts to suicidal behavior.

Veterinarians are exposed to a range of occupational stressors that contribute to higher rates of mental health problems and suicide risk compared to the general population (10, 14, 15) These stressors include chronic work overload, economic instability, and frequent engagement in emotionally demanding tasks such as euthanasia and supporting grieving clients. Together, these factors may increase the risk of depression, anxiety, and professional burnout, all of which are associated with suicidal behavior (11, 16, 17).

Among the most emotionally demanding responsibilities in veterinary practice is euthanasia. Although euthanasia may be medically and ethically justified, it can also generate moral stress arising from conflict between professional duties, including euthanasia, and personal values (13, 13, 18, 19). Moral stress is related to, but distinct from, moral distress and moral injury. As Buchbinder et al. (20) note, moral stress can emerge from the normal functioning of overstressed systems and may be pervasive and chronic.; Unlike moral distress, it does not necessarily involve a strong sense of powerlessness, and unlike moral injury, it does not require a discrete transgressive event. This distinction is particularly relevant in veterinary medicine, where repeated euthanasia within an under-resourced system may generate chronic, systems-level moral burden. Although, Burnout, ethical dilemmas, and moral stress, are related but distinct constructs- research indicates that repeated involvement in euthanasia is associated with higher levels of secondary traumatic stress, burnout and ethical distress (18, 21).

In Poland, this burden may be intensified by the predominantly private organization of the veterinary sector and the limited availability of institutional after-hours emergency care. As a result, many practitioners are expected to remain on call or provide urgent services beyond standard working hours. This structure may contribute to chronic overload, reduced work-life balance, and professional isolation, thereby amplifying psychological strain and vulnerability to burnout. The ethical dilemmas involved in balancing animal welfare, client expectations, and professional judgment may further intensify this burden (13).

Another distinctive occupational risk factor is veterinarians’ access to lethal substances such as pentobarbital, commonly used in euthanasia procedures. Studies have shown that a substantial proportion of suicides among veterinarians involve euthanasia drugs, often obtained from their workplace (11, 22). Importantly, when deaths due to pentobarbital poisoning are excluded from statistical analyses, the suicide rate among veterinarians is no longer significantly elevated compared to the general population (22). This finding highlights the potentially critical role of access to lethal means in shaping suicide risk in this profession.

Similar patterns have been observed in other occupations with access to lethal agents. In a systematic review of anesthetists, Plunkett et al. (2021) (23) reported a drug-related suicide ratio of 2.21 (95% CI 1.33–3.66) compared with comparator groups, attributing this in part to easy access to and knowledge of potent anesthetic drugs such as propofol. Among pharmacists, age-adjusted suicide rates have been also reported to exceed those of the general population (24). Across healthcare professions more broadly, Dutheil et al. (2019) (25) found an overall standardized mortality ratio for suicide in physicians of 1.44 (95% CI 1.16–1.72), with female physicians at particularly elevated risk (SMR = 1.94). Together, these findings support the interpretation of occupational access to lethal means as a profession-specific suicide risk factor.

Although awareness of these risks is increasing, a more integrated understanding of how moral stress, euthanasia practices, and access to lethal substances interact to influence suicide risk among veterinarians is still lacking. Previous research in veterinary practitioners has largely considered these elements in isolation, and their combined effects remain underexplored. A better understanding of these relationships may inform targeted prevention strategies, including accessible mental health support, ethical training, and stricter protocols governing access to euthanasia drugs. (11, 16, 22). A similar need in terms of more comprehensive models exists in suicide research more broadly. A meta-analysis of 365 studies from the past 50 years found that prediction of STBs was only slightly better than chance across all outcomes and methods, that no single category of risk factors consistently stood out, and the predictive accuracy had not improved over time (3). The authors argued that these findings support a shift from traditional risk-factor approaches toward machine learning-based risk algorithms (3).

This study addresses both gaps through a nationwide cross-sectional survey of Polish veterinarians. Using validated psychometric tools and advanced analytical methods, including machine learning and network analysis, we sought to identify psychological, clinical, social, and occupational predictors of suicide-related outcomes, with particular emphasis on whether occupational access to euthanasia drugs and repeated exposure to assisted death may contribute to suicide risk in this population.

Based on the theoretical framework and literature outlined above, we formulated the following hypotheses. First (H1), given the established elevated rates of psychological distress, burnout, and suicidal ideation among veterinary professionals documented in prior international research, we hypothesized that a substantial proportion of the Polish veterinary sample would score above clinical cut-off thresholds on measures of suicidal behavior and acquired capability for suicide, reflecting a level of risk meaningfully exceeding that expected in the general population.

Second (H2), drawing on the Three-Step Theory and the ideation-to-action distinction, and given that the ACWRSS includes items capturing mental rehearsal and method-specific thinking that map directly onto occupational familiarity with euthanasia, we hypothesized that euthanasia-related cognitions would show stronger associations with acquired capability for suicide — particularly its rehearsal and preparation subscale — than with suicidal behavior history, and would be independently associated with both outcomes over and above general psychopathological variables in regression models.

Third (H3), given that moral stress represents a contextual occupational stressor arising from the conflict between professional duties and personal values rather than a clinical risk factor, we hypothesized that it would show a meaningful association with suicidal behavior and acquired capability for suicide, but that this association would be weaker than that of general psychological distress variables such as depression, anxiety, and burnout.

## 2 Methods

The Mental Health of Veterinary Doctors (MEDWET) study was initiated by the National Veterinary Medical Council to identify individual, social, and environmental risk factors affecting the mental health of veterinary doctors in Poland. The study also aimed to assess the specific needs of this professional community. The MEDWET study was designed as an anonymous nationwide online survey. The online format enabled broad reach while preserving participant anonymity.

Ethical approval was obtained from the Bioethical Committee of the Hirszfeld Institute of Immunology and Experimental Therapy, Polish Academy of Sciences (No. 5/2023). The study was conducted in accordance with the Declaration of Helsinki.

### 2.1 Participants and data collection

To participate, respondents were required to provide informed consent for anonymous completion of the online survey and confirm that they were practicing veterinary doctors in Poland. No additional exclusion criteria were applied.

The final sample consisted of 783 respondents. For context, the total number of registered veterinarians in Poland is approximately 22 000. Data collection was conducted nationwide between April 2023 and February 2024, using the Computer-Assisted Web Interviewing (CAWI) method. This approach allowed for broad participant reach while ensuring convenience and anonymity, which encouraged respondents to share candid insights, even on sensitive personal topics. The survey was distributed through the contact database of the National Veterinary Medical Council and through closed social media groups for veterinary professionals.

### 2.2 Survey development

The final version of the online survey was developed through a multi-step process. First, a literature review was conducted using scientific journal databases including PubMed, Google Scholar, to identify relevant topics and risk factors associated with the mental health of veterinary doctors. Second, regular in-person and online consultations were held with members of the Committee for Media Policy and Internal Communication of the National Veterinary Medical Council (KRLW). These representatives, drawn from various veterinary professional groups, provided insights into the specific challenges and characteristics of the profession. Third, after developing the initial questionnaire draft a pilot validation phase was conducted among Committee members. Their qualitative and quantitative feedback was reviewed, discussed, and incorporated into the final survey version.

### 2.3 Measures

The survey included a range of standardized psychometric instruments assessing psychological, social, clinical, and occupational domains.

Psychological scales were used to assess burnout and resilience the Maslach Burnout Inventory (MBI), which consists of 22 questions and measures three main components of occupational burnout: Emotional Exhaustion, Depersonalization and Low Personal Accomplishment (Cronbach’s α calculated for the study group was= 0.889) and the Brief Resilience Coping Scale (BRS), which consists of four questions that measure how well a person can cope with challenges and bounce back from difficult situations (Cronbach’s α= 0.733).

Social aspects were assessed using the Multidimensional Scale of Perceived Social Support (MSPSS), which consists of 12 questions and measures support in three dimensions: Family Support, Friend Support and Significant Other Support (Cronbach’s α= 0.944).

General psychopathology was measured with the use of the General Health Questionnaire-28 (GHQ-28), which is a psychometric tool designed to assess overall mental health. It consists of 28 questions, divided into four subscales, each evaluating a different aspect of mental well-being: Somatization, Anxiety and Insomnia, Social Dysfunction and Depression (Cronbach’s α= 0.952). Depressive symptoms were assessed using the The Patient Health Questionnaire-9 (PHQ-9) which consists of nine questions, which align with the diagnostic criteria for depression according to the DSM-IV. Each question evaluates the frequency of specific symptoms over the past two weeks (Cronbach’s α= 0.91). Anxiety symptoms were assessed using the General Anxiety Disorder (GAD-7), which consists of seven questions, each evaluating the frequency of specific anxiety symptoms over the past two weeks (Cronbach’s α= 0.947). Additionally, The Alcohol, Medication, and Drug Use Questionnaire (AMDQ) was used as a tool assessing patterns and effects of psychoactive substance use in individuals (Cronbach’s α= 0.761).

STBs were assessed with the Acquired Capability with Rehearsal for Suicide Scale (ACWRSS), which measures two main aspects: Acquired capability for suicide — involves a gradual adaptation to pain (Pain Tolerance, ACWRSS_PT) and fear associated with suicide (Fearlessness of Death, ACWRSS_FOD) due to previous traumatic experiences or self-injury, and Suicidal preparation attempts — assesses thoughts and behaviors related to planning or attempting suicide (Preparation for Suicide, ACWRSS PFS), such as imagining suicide or mimicking suicidal actions (Cronbach’s α= 0.646) as well as the Suicidal Behaviors Questionnaire-Revised (SBQ-R), which consists of four questions, covering: History of Suicidal Thoughts (lifetime suicidal ideation or attempts, SBQ LTP), Frequency of Suicidal Thoughts, Disclosure of Suicidal Intent and Suicide Attempts (Cronbach’s α= 0.787).

In addition to the standardized psychometric questionnaires, the survey included several self-constructed composite indices and single-question items designed to capture occupational and suicide-related experiences specific to veterinary practice.

OW (Overworked) was developed as a composite index of the subjective work overload (Cronbach’s α = 0.756), It included five dichotomized items reflecting time pressure, excessive workload, diminished autonomy, intention to leave the profession, and lack of time for personal interests. Scores ranged from 0 to 5, with higher scores indicating greater perceived work overload.

WRN (Work-Related Neglect) measures occupational self-neglect(Cronbach’s α =0.756). It was derived from seven dichotomous items assessing the frequency of neglecting sleep, nutrition, health, and personal care due to work. Scores ranged from 0 to 7, with higher scores indicating greater self-neglect.

SAW (Support at Work) assessed perceived workplace support (Cronbach’s α = 0.559). It includes five items evaluating emotional, instrumental, and supervisory support in the work environment. Scores ranged from 0 to 5, with higher values indicating greater perceived support.

IR TOTAL (Interpersonal Relationships) represented the relationship strain index (Cronbach’s α= 0.773). This composite score reflects challenges in interpersonal relationships, based on responses to four items (R2–R5). The questions assess tension, conflict, and communication difficulties in both professional and personal contexts. The total score ranges from 0 to 4, with higher scores indicating greater relational difficulties.

SM measured moral stress related to euthanasia using the item:: “Do you experience so-called moral stress when performing euthanasia on an animal?” This variable was intended to capture emotional or ethical distress associated with euthanasia.

Suicide exposure was assessed with three single-item variables: S2,exposure to suicide in the professional environment: “*Did you know any people in your professional environment who died by suicide?*”. S3, umber of known suicide deaths: And S4, nature of relationship to the deceased.

For S4, response options ranged from a close personal relationship to no personal relationship and were coded to reflect relational proximity.

E3 assessed thoughts about using euthanasia drugs on oneself (*“Have you ever thought about using euthanasia drugs on yourself?”).* Responses are coded dichotomously (Yes/No). As a single-item self-report measure without external psychometric validation, E3 should be interpreted as an exploratory indicator of crisis-related ideation rather than a validated clinical construct.

Unless otherwise specified above, self-constructed indices referred to respondents’ current experiences. These items were developed based on a literature review of factors associated with the mental health in veterinary proprofessionals (e.g. 10, 12, 16, 21, 26–36) and were refined during the expert consultation and pilot-testing stages described above.

Place of residence and financial independence were included as demographic descriptors because of their potential relevance to professional isolation and economic vulnerability in the Polish veterinary context.

### 2.4 Data analysis

Descriptive statistics (mean, standard deviation, and frequency distributions) were computed for all suicide-related variables. To evaluate gender differences, independent samples t-tests were conducted comparing scores between women and men, excluding the small number of non-binary participants from inferential analysis due to limited statistical power. To examine age-related differences, participants were categorized into six age groups: <30, 30–39, 40–49, 50–59, 60–69, and 70+. One-way analyses of variance (ANOVAs) were performed to assess between-group differences across these age categories for each suicide-related variable. Effect sizes were reported as Cohen’s d for t-tests and η² for ANOVAs. Statistical significance was set at *p* <.05 for all tests. Missing data were handled pairwise for descriptive and inferential analyses. A missing data analysis indicated that 95.7% of participants (750/783) had complete data on all key variables; the exception was SM2 (moral stress), which had 34 missing cases (4.3%), likely attributable to item sensitivity. Only one participant had missing data on more than one key variable. No systematic pattern of missingness was identified. Regression analyses were conducted on complete cases (N = 750). A *post-hoc* power analysis indicated that the regression models were well-powered: the SBQ model (R²=.467, f²=0.876) and the ACWRSS model (R²=.220, f²=0.282) both exceed conventional thresholds for large effects, yielding estimated power >.99 at α=.05 with N = 750 for both models. Variance Inflation Factors (VIF) were inspected for all predictors; the maximum observed VIF was 3.51 (GHQ-28), well below the threshold of 10, indicating no meaningful multicollinearity. Both standardized and unstandardized beta coefficients were reported to facilitate interpretation and comparison of relative predictor magnitude.

### 2.5 Machine learning-based variable importance analysis

Using machine learning methods (random forests), a hierarchy of importance for independent variables was created for each dependent variable. This was executed using Feature Importance, Gini Importance, and Total Decrease in Node Impurity. These methods allowed us to determine which independent variables have the greatest impact on each dependent variable. This methodology is widely recognized for its high accuracy and efficiency, resistance to overfitting, and ability to handle large datasets, making it highly popular in data analysis. Both numerical and categorical variables were analyzed.

A separate Network Analysis was then conducted to examine interconnected relationships between the dependent and independent variables and their dependency structure. Partial correlations were used to describe these relationships within the network. The analyses were performed using the following software: Matlab R2024b, R 4.4.2, SPSS and Python 3.10.10.

## 3 Results

Results are presented in four sections. First, descriptive statistics on the study demographic are presented (3.1), second descriptive statistics and group differences in suicide-related variables are reported, including sample characteristics (3.2). Second, machine learning feature importance and network analysis findings are presented (3.3). Third, multiple linear regression models predicting suicidal behavior and acquired capability for suicide are reported (3.4).

### 3.1 Sample characteristics

The final study sample included 783 respondents. The mean age of participants was 39.4 years (*SD* = 10.7), ranging from 24 to 84 years. The 25th, 50th (median), and 75th percentiles were 31, 37, and 45 years, respectively. Women constituted 63.6% of the sample (n=498), while 0.6% (n=5) identified as other/non-binary. A detailed description of the study group in terms of gender, source of income, place of residence can be found in **Table 1**. In terms of professional experience, the respondents had an average of 13.6 years of work in the field (*SD* = 10.6; range: 0.08–63 years). The most frequent work settings included private practice, public veterinary inspection services, pharmaceutical industry, laboratories, and academic/research institutions, with many individuals working across multiple domains. Respondents reported working an average of 9.2 hours per day (*SD* = 5.6), and 6.6 days per week (*SD* = 6.3), with some outliers working exceptionally long hours or days.

**Table 1:** Sample characteristics (N = 783).

Demographic variable	n	%
Gender		
Female	498	63.6
Male	280	35.8
Other/Non-binary	5	0.6
Place of residence		
Large city (>300,000)	267	34.1
Medium city (100,000–300,000)	184	23.5
Small town (<100,000)	144	18.4
Village	132	16.9
Other	56	7.2
Main source of income		
Jointly with partner	401	51.2
Independently	286	36.5
Other	96	12.3
Relationship status		
Married/in partnership	646	82.5
Single/divorced/widowed	137	17.5

### 3.2 Suicide risk

The analysis of suicide-related variables revealed a concerning prevalence of suicide risk factors among the surveyed group. The ACWRSS showed a mean total score of 22.06 (SD = 11.69). Subcomponents of this scale show variability, with Pain Tolerance averaging 5.54, Fearlessness of Death at 8.25, and Preparation for Suicide at 8.28.

The SBQ-R further highlights risk. The total score averaged 6.56 (SD = 3.48), with 41.6% of respondents scoring at or above the clinical cut-off of ≥7, suggesting elevated suicide risk in nearly half the sample. Additionally, the SBQ LTP score, averaged 1.86, indicating that many individuals have seriously considered or attempted suicide at some point. Approximately 58% (SBQ LTP cut-off of ≥2) reported at least one such experience.

In terms of exposure to suicide (S2), 55.2% of respondents reported knowing at least one person in their professional environment who had died by suicide. Among those, the average number of known suicide deaths (S3) was 2.39. The nature of these relationships (S4) varied but tended to be professionally close, with averaging 1.38 on a scale reflecting relational closeness. 23.7% of participants reported having thoughts about using euthanasia drugs on themselves (E3).

No statistically significant associations were found between knowing someone in the professional environment who died by suicide (S2), the number of such individuals (S3), or the nature of the relationship (S4) and SBQ-R scores or ACWRSS total scores.

#### 3.1.1 Gender and age differences in suicide-related risk factors

Statistical analysis revealed significant gender- and age-related differences in suicide-related risk factors. Men reported higher levels of ACWRSS FOD than women, contributing to a higher PFS. In contrast, women exhibited significantly higher suicidal total risk scores on the SBQ-R, suggesting they are more likely to experience suicidal ideation or engage in related behaviors. Detailed results of the gender comparisons can be found in **Table 2**.

**Table 2:** Results of independent samples T-tests comparing suicide-related variables by gender (female vs. male).

Variable	M female (SD)	M male (SD)	t-statistic	p-value	d
ACWRSS TOTAL	21.76 (11.98)	22.63 (11.20)	-1.037	0.3	-0.074
ACWRSS PT	5.35 (4.62)	5.88 (4.75)	-1.49	0.137	-0.113
ACWRSS FOD	7.67 (4.80)	9.30 (4.88)	**-4.494**	< 0.001***	-0.337
ACWRSS PFS	8.74 (8.57)	7.45 (7.69)	**2.108**	0.035*	0.156
SBQ-R	6.78 (3.54)	6.14 (3.33)	**2.461**	0.014*	0.184
SBQ-R cut off >7	0.44 (0.50)	0.36 (0.48)	**2.128**	0.034*	0.160
SBQ LTP	1.93 (0.89)	1.73 (0.83)	**2.993**	0.003**	0.222
SBQ LTP cut-off ≥ 2	0.61 (0.49)	0.52 (0.50)	**2.402**	0.017*	0.184
S1 -S4, E3, SM	—	—	All n.s.	>0.5	all <|0.06|

*P-values *p < 0.05, **p < 0.01, ***p < 0.001.

M, mean; SD, standard deviation; d, Cohen's d. ACWRSS, Acquired Capability with Rehearsal for Suicide Scale; PT, Pain Tolerance; FOD, Fearlessness of Death; PFS, Preparation for Suicide; SBQ-R, Suicidal Behaviours Questionnaire-Revised; LTP, lifetime suicidal ideation and/or attempt; S1–S4, suicide exposure items; E3, thoughts about using euthanasia drugs on oneself; SM, moral stress. Bold values indicate statistical significance at p < 0.05. Asterisks denote the level of significance: *p < 0.05, **p < 0.01, ***p < 0.001.

Age group comparisons using ANOVA further underscore these patterns. Younger participants (<30 years) reported the highest levels of suicide ideation and behavior (SBQ-R) and preparation for suicide (ACWRSS PFS). As age increased, overall suicide risk and capability scores declined. Notably, fearlessness of death was the only component that increased with age, peaking in the oldest group (70+), although their overall risk remained the lowest. For detailed results see **Table 2**. The analysis of moral stress (SM), which reflects the emotional burden associated with performing euthanasia on animals, revealed a statistically significant difference across age groups (*F*(5, 741)=3.53, *p* = .0037). *Post hoc* comparisons using the Tukey HSD test indicated that respondents aged 60–69 reported significantly higher levels of moral stress compared to those aged 30–39 (*p* = .016). No other age group comparisons reached statistical significance.

### 3.2 Feature importance and network analysis

**Figure 1:**
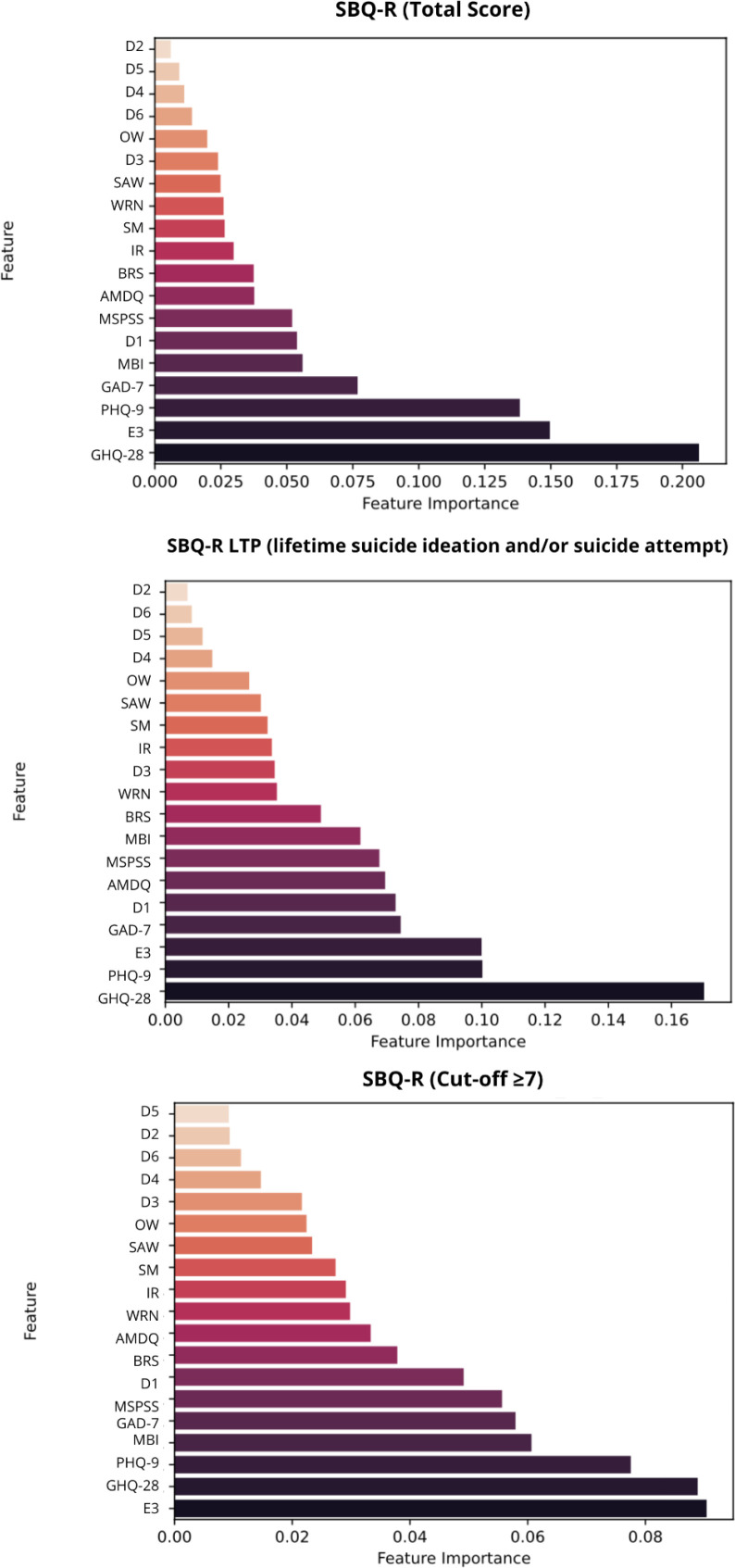
SBQ-R total score and subscores – feature importance analysis. D1, Age; D2, Gender; D3, Place of residence; D4 – Source of income; D5, Marital status; D6, Children; OW, Overworked; WRN, Work-Related Neglect (total score); SAW, Support At Work; MBI, Occupational Burnout (Maslach Burnout Inventory, total score); MSPSS, Multidimensional Scale of Perceived Social Support (total score); IR, Interpersonal Relationships (total score); BRS, Brief Resilience Scale (total score); SM, Moral Stress; GHQ-28, General Health Questionnaire (total score); PHQ-9, Patient Health Questionnaire-9 (total score); GAD-7, Generalized Anxiety Disorder-7 (total score); AMDQ, Alcohol/Medication/Drug Use Questionnaire (total score); E3, Have you ever thought about using animal euthanasia drugs on yourself?

**Figure 2:**
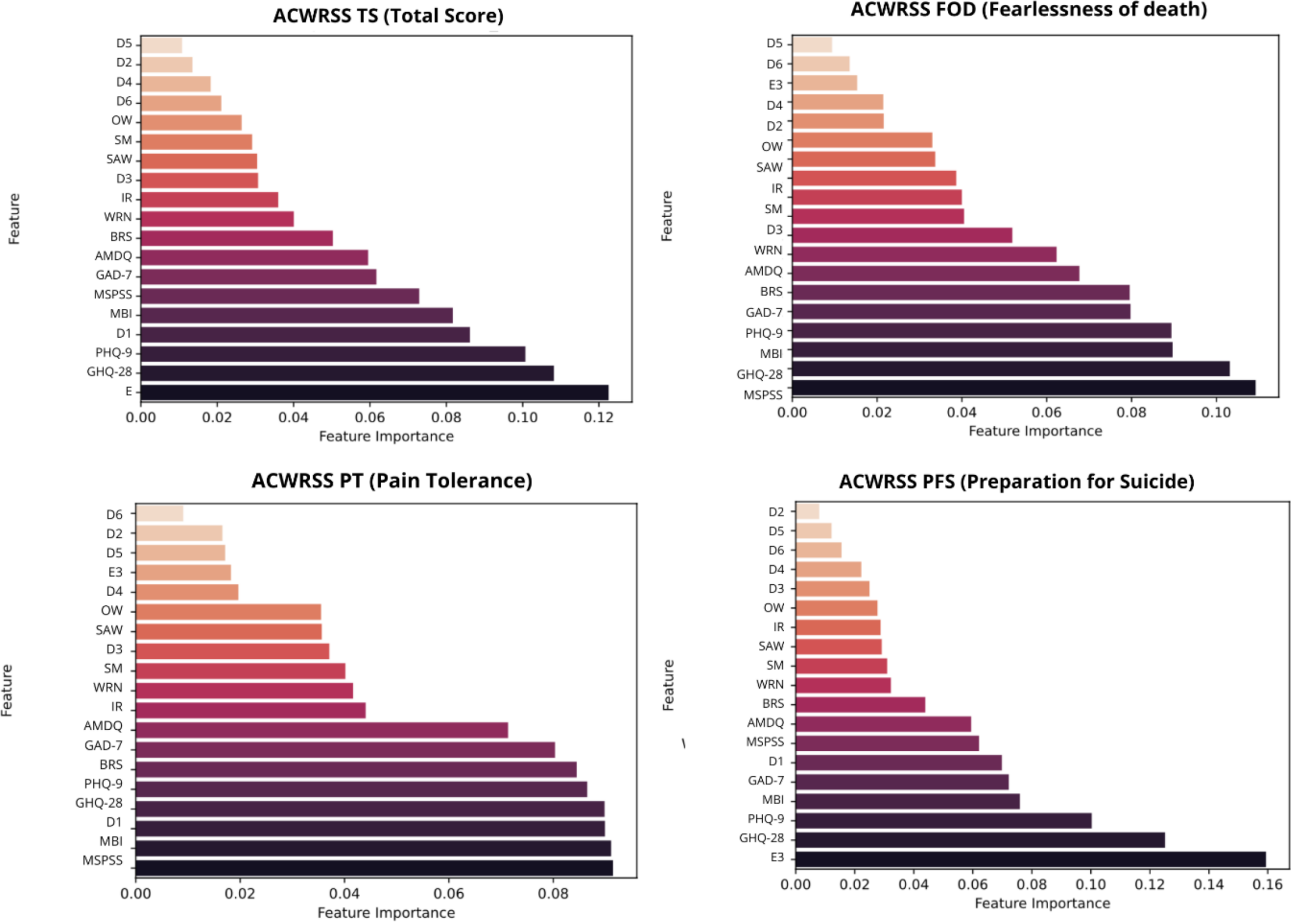
ACWRSS total score and subscores – feature importance analysis. D1, Age; D2, Gender; D3, Place of residence; D4, Source of income; D5, Marital status; D6, Children; OW, Overworked; WRN, Work-Related Neglect (total score); SAW, Support At Work; MBI, Occupational Burnout (Maslach Burnout Inventory, total score); MSPSS, Multidimensional Scale of Perceived Social Support (total score); IR, Interpersonal Relationships (total score); BRS, Brief Resilience Scale (total score); SM, Moral Stress; GHQ-28, General Health Questionnaire (total score); PHQ-9, Patient Health Questionnaire-9 (total score); GAD-7, Generalized Anxiety Disorder-7 (total score); AMDQ, Alcohol/Medication/Drug Use Questionnaire (total score); E3, Have you ever thought about using animal euthanasia drugs on yourself?

Random forest models were applied to identify the most influential predictors for a range of dependent variables related to suicidality and psychological risk in veterinary professionals. Variables considered included demographic, occupational, psychological, and clinical characteristics, that were determined to correlate with suicidal risk based on previous literature as well as exploratory correlational analyses of the obtained data. The analysis identified item E3, a self-reported item assessing whether the respondent had ever considered using euthanasia drugs on himself, as the strongest predictor of ACWRSS total scores. Item E3 also emerged as the dominant predictor for the ACWRSS_PFS, far outweighing all other variables. Other important predictors included GHQ-28, PHQ-9, and D1. For ACWRSS_PT the most influential predictors were MSPSS, MBI, GHQ-28 and D1. The ACWRSS subscale FOD was predicted primarily by D1. Interestingly, item E3 had no significant impact on these latter two dimensions. The SBQ-R was primarily predicted by the three main variables — E3, GHQ-28 and PHQ-9 with a different order of relevance. SM was not among major contributors, when all variables were included in the model - placing in the lower half of all of the variables. Visual representations of these results are provided in **Figure 1** and **Figure 2** illustrating the hierarchy of predictor importance for each outcome.

A regularized partial correlation network was constructed to explore the structural relationships among dependent and independent variables. Each node represents a variable, and edges represent partial correlations, controlled for all other variables in the network. Edge weight (thickness and color intensity) corresponds to the strength of the direct association, with blue indicating positive and red indicating negative correlations.

**Figure 3:**
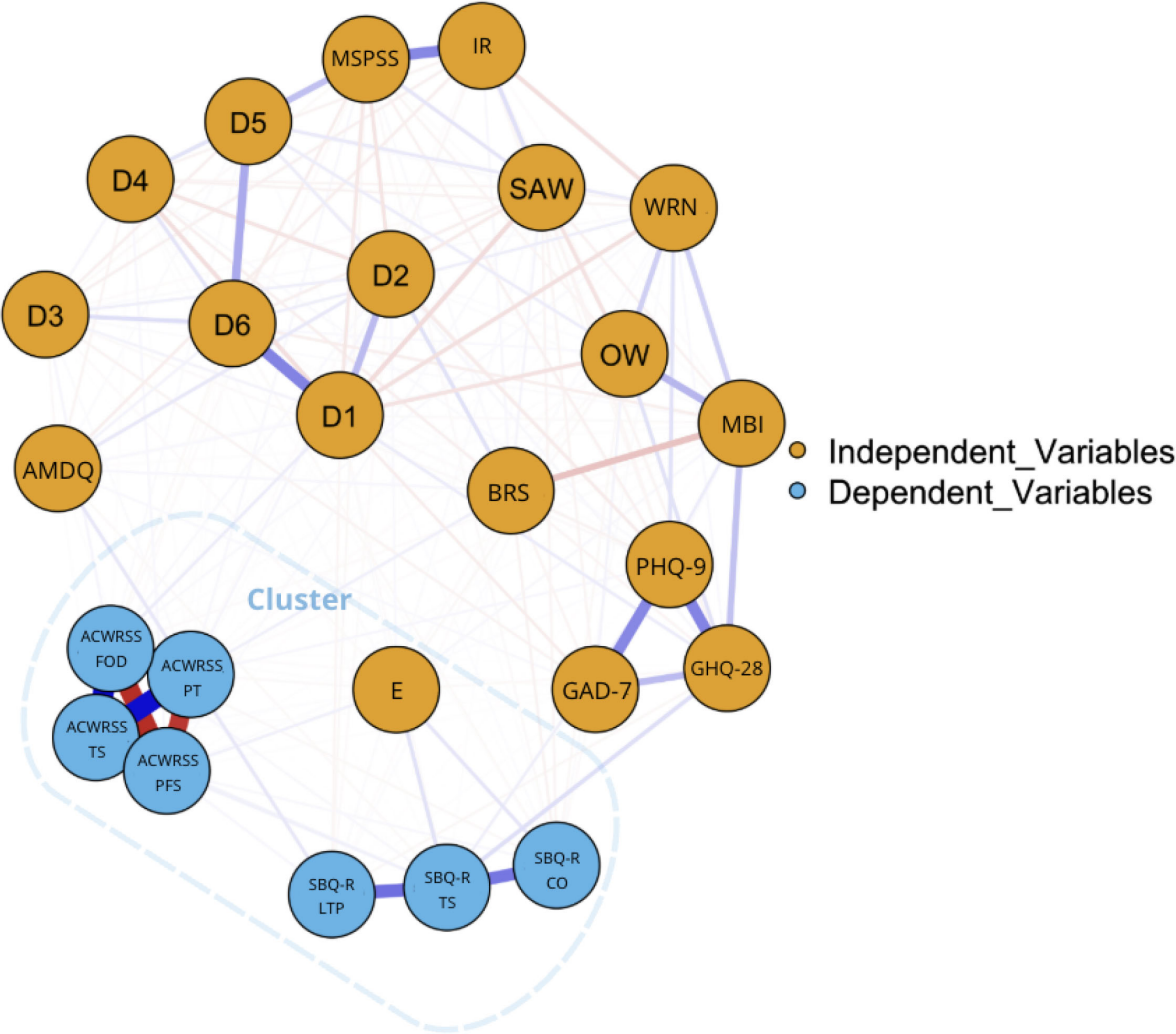
Network analysis with cluster analysis. D1, Age; D2, Gender; D3, Place of residence; D4, Source of income; D5, Marital status; D6, Children; OW, Overworked; WRN, Work-Related Neglect (total score); SAW, Support At Work; MBI, Occupational Burnout (Maslach Burnout Inventory, total score); MSPSS, Multidimensional Scale of Perceived Social Support (total score); IR, Interpersonal Relationships (total score); BRS, Brief Resilience Scale (total score); GHQ-28, General Health Questionnaire (total score); PHQ-9, Patient Health Questionnaire-9 (total score); GAD-7, Generalized Anxiety Disorder-7 (total score); AMDQ, Alcohol/Medication/Drug Use Questionnaire (total score); E, Have you ever thought about using animal euthanasia drugs on yourself?; ACWRSS TS, Acquired Capability with Rehearsal for Suicide Scale, total score; ACWRSS PT, Acquired Capability with Rehearsal for Suicide Scale, Pain Tolerance; ACWRSS FOD, Acquired Capability with Rehearsal for Suicide Scale, Fearlessness of Death; ACWRSS PFS, Acquired Capability with Rehearsal for Suicide Scale, Preparation for Suicide; SBQ-R TS, Suicide Behaviors Questionnaire-Revised, total score; SBQ-R CO, Suicide Behaviors Questionnaire-Revised, clinical cut-off ≥7; SBQ-R LTP, Suicide Behaviors Questionnaire-Revised, lifetime suicide ideation and/or suicide attempt.

Key findings from the network (**Figure 3**) include: Variables from the ACWRSS and SBQ-R domains formed a tightly interconnected cluster, indicating strong interrelations among different dimensions of suicidality. Item E3 was located at the center of this suicidality cluster, despite having low overall centrality, suggesting its specific and targeted relevance to suicide-related variables. Strong connections were also observed between suicidality measures and GHQ-28, PHQ-9, GAD-7, AMDQ, BRS, and Age. A Cluster Analysis (**Figure 3**) confirmed the unique role of item E3 as a bridge between ACWRSS and SBQ-R outcomes, further validating its centrality in occupational suicide risk. SM does not cluster with the central suicide-related variables but maintains clear connections to core psychological and demographic variables, including D1 (Age) and other independent measures.

Three centrality metrics were calculated. Strength was highest for ACWRSS variables, indicating their strong direct connections with other nodes. Betweenness and Closeness were highest for D1, positioning it as a key connector and potentially influential variable across the network. Item E3, while crucial in the Feature Importance analyses, exhibited low centrality in the network model, suggesting that its influence is concentrated on specific suicidality variables and not widespread across the broader psychosocial landscape (**Figure 3**).

### 3.3 Multiple linear regression analysis

A multiple linear regression was conducted to identify predictors of suicidal behavior — SBQ-R (**Table 3**). The overall model was statistically significant, F(11, 738)=58.68, p<.001, explaining 46.7% of the variance in SBQ-R total scores (Adjusted R²= .459). The analysis was conducted on N = 750 complete cases. Variance Inflation Factors (VIF) for all predictors ranged from 1.04 to 3.51, indicating no multicollinearity concerns. Significant predictors of suicidal behavior included: GHQ-28 (β=0.091,β*=0.453, p<.001), indicating higher psychological distress predicted greater suicidality, E3 (β=2.83, β*=0.350, p<.001), representing a large increase in suicidality among those reporting thoughts on using euthanasia drugs on themselves, Age (β=–0.039, β*=−0.121, p<.001), where younger age was associated with greater suicidality, MSPSS (β=–0.013, β*=−0.068, p= .040), with greater perceived social support linked to lower suicidality as well as AMDQ (β=0.034, β*=0.057, p= .037), indicating that greater substance use was associated with higher suicidality. Other predictors, including anxiety, burnout, resilience, interpersonal functioning, moral stress, and work-related neglect, were not statistically significant in the presence of the stronger predictors. Another multiple linear regression analysis was conducted to examine whether psychological, occupational, and interpersonal variables predicted acquired capability for suicide (**Table 4**). The overall model was statistically significant, F(12, 737)=17.34, p<.001, and accounted for approximately 22.0% of the variance in the ACWRSS total score (Adjusted R²= .208). The analysis was conducted on N = 750 complete cases; VIF ranged from 1.04 to 3.51. Three predictors emerged as statistically significant: GHQ-28 (β=0.22, β*=0.323, *p* <.001), indicating that greater psychological distress was associated with higher acquired capability for suicide; E3 (β=8.58, β*=0.315, *p* <.001), suggesting that those who had considered using euthanasia drugs on themselves scored nearly nine points higher on ACWRSS; and AMDQ (β=0.15, β*=0.072, *p* = .030), indicating that higher substance use was associated with increased capability for suicide. Age and resilience (BRS) approached statistical significance (*p* = .081 and *p* = .063, respectively), while all other predictors were non-significant (all *p*>.10). These findings suggest that psychological distress, suicidal ideation involving euthanasia methods, and substance use are key contributors to acquired capability for suicide in this sample, while work-related and relational factors showed no significant independent associations in the full model.

**Table 3:** One-way ANOVA results for age group differences in suicide-related variables.

Variable	F-statistic	p-value	η²
ACWRSS TOTAL	3.254	0.006**	.021
ACWRSS_PT	2.868	0.014**	.018
ACWRSS_FOD	5.823	< 0.001***	.036
ACWRSS_PFS	8.4	< 0.001***	.052
SBQ-R	8.962	< 0.001***	.055
SBQ-R cut-off ≥ 7	6.916	< 0.001***	.043
SBQ LTP	9.251	< 0.001***	.056
SBQ LTP cut-off ≥ 2	10.835	< 0.001***	.065
S2	5.731	< 0.001***	.036
S4	3.235	0.007**	.020
SM	3.527	0.003	.023
S1, S3, E3 (n.s.)	—	>.05	<.010

*P-values *p < 0.05, **p < 0.01, ***p < 0.001.

ACWRSS, Acquired Capability with Rehearsal for Suicide Scale; PT, Pain Tolerance; FOD, Fearlessness of Death; PFS, Preparation for Suicide; SBQ-R, Suicidal Behaviours Questionnaire-Revised; LTP, lifetime suicidal ideation and/or attempt; S1–S4, suicide exposure items; E3, thoughts about using euthanasia drugs on oneself; SM, moral stress.

**Table 4:** Multiple linear regression analysis predicting suicidal behavior (SBQ-R total score) and acquired capability for suicide (ACWRSS total score).

Predictor	Outcome variable	Estimate (β)	Std. Error	p-value	β*	VIF
*SBQ-R Total Score*	**Intercept**	**6.074**	**1.107**	< 0.001***	—	—
	**GHQ-28**	**0.091**	**0.01**	< 0.001***	0.453	3.51
	**E3**	**2.828**	**0.226**	< 0.001***	0.350	1.08
	GAD-7	0.005	0.024	0.832	0.010	2.87
	MBI	-0.013	0.013	0.3	-0.042	2.28
	**Age**	**-0.039**	**0.01**	< 0.001***	-0.121	1.23
	**MSPSS**	**-0.013**	**0.006**	0.04*	-0.068	1.52
	**AMDQ**	**0.034**	**0.016**	0.037*	0.057	1.04
	BRS	-0.037	0.033	0.259	-0.036	1.41
	IR	0.03	0.053	0.58	0.019	1.58
	SM	0.041	0.058	0.481	0.020	1.10
	WRN	-0.02	0.061	0.735	-0.011	1.52
*ACWRSS Total Score*	**Intercept**	**14.492**	**4.764**	0.002**	—	—
	**GHQ-28**	**0.219**	**0.041**	< 0.001***	0.323	3.51
	**E3**	**8.582**	**0.923**	< 0.001***	0.315	1.08
	GAD-7	-0.129	0.101	0.199	-0.071	2.89
	MBI	-0.033	0.052	0.534	-0.031	2.29
	Age	-0.07	0.04	0.081	-0.064	1.29
	MSPSS	-0.033	0.026	0.206	-0.051	1.54
	**AMDQ**	**0.146**	**0.067**	0.03**	0.072	1.04
	BRS	0.248	0.133	0.063	0.072	1.41
	IR	-0.046	0.22	0.835	-0.009	1.61
	SM	0.334	0.238	0.161	0.048	1.10
	WRN	0.086	0.248	0.728	0.014	1.53
	SAW	0.377	0.336	0.262	0.041	1.26

*P-values *p < 0.05, **p < 0.01, ***p < 0.001.

β, unstandardised coefficient; β*, standardised coefficient (NEW); SE, standard error; VIF, Variance Inflation Factor (NEW). ACWRSS, Acquired Capability with Rehearsal for Suicide Scale; SBQ-R, Suicidal Behaviours Questionnaire-Revised; GHQ-28, General Health Questionnaire; E3, thoughts of using euthanasia drugs on oneself; MBI, Maslach Burnout Inventory; MSPSS, Multidimensional Scale of Perceived Social Support; AMDQ, Alcohol/Medication/Drug Questionnaire; BRS, Brief Resilience Scale; IR, Interpersonal Relationships; SM, Moral Stress; WRN, Work-Related Neglect; SAW, Support at Work. Bold values indicate statistical significance at p < 0.05. Asterisks denote the level of significance: *p < 0.05, **p < 0.01, ***p < 0.001.

## 4 Discussion

The present findings provide differential support for the study hypotheses. First (H1), the hypothesis that a substantial proportion of the sample would exceed clinical thresholds for suicide risk and acquired capability was clearly supported, with nearly half of participants scoring above the SBQ-R cut-off and elevated ACWRSS scores observed across the sample. Second (H2), euthanasia-related cognitions emerged as a strong and independent predictor of both suicidal behavior and acquired capability, even after controlling for general psychopathology, thus partially supporting the hypothesis. Although the association appeared numerically stronger for acquired capability, consistent with the ideation-to-action framework, this difference was not formally tested and should be interpreted with caution. Third (H3), the hypothesis that moral stress would be associated with suicidality, albeit more weakly than general psychological distress, was not supported in regression models, where moral stress did not emerge as a significant independent predictor. However, its associations with age and its positioning within the broader psychosocial network suggest a more indirect or contextual role, indicating that moral stress may contribute to vulnerability through pathways not captured by direct effects on suicidality outcomes.

The current study significantly advances our understanding of the complex factors contributing to elevated suicide risk among veterinary professionals. Two distinct aspects of suicidality were assessed in this study: suicidal thoughts and behaviors using the Suicidal Behaviors Questionnaire–Revised (SBQ-R), and capability for suicide using the Acquired Capability with Rehearsal for Suicide Scale (ACWRSS). While the SBQ-R captures an individual’s subjective history and frequency of suicidal ideation, disclosure of suicidal intent, and past suicide attempts, the ACWRSS focuses on the psychological and behavioral capacity to enact suicide, including both acquired tolerance to pain and fear as well as rehearsed preparatory behaviors such as imagining or simulating suicide. Consistent with prior international research (10, 11, 37–39), our findings confirm that veterinarians exhibit a high prevalence of suicide-related ideation and behaviors, underscored by elevated scores on suicide assessment scales like the SBQ-R and ACWRSS.

Our results demonstrate that both suicidal behavior and the acquired capability for suicide are strongly associated with psychological distress, depressive symptoms, and burnout. These findings are consistent with prior studies indicating that veterinarians experience higher levels of depression, burnout and anxiety than the general population (11, 12, 40). For Suicide behaviors the strongest predictor was general psychological distress, followed by depressive symptoms and thoughts about using euthanasia drugs on themselves. These results suggest that both general and mood-related psychopathology, along with access to euthanasia methods, strongly associated with suicidal thoughts and behaviors in this population. The relationships support earlier findings that observed psychopathological symptoms are central predictors of suicidal ideation (10).

Gender differences observed in this study align broadly with existing literature (12, 39). Women reported higher overall suicidality, while men demonstrated higher levels of acquired capability for suicide, particularly in terms of fearlessness of death. Effect sizes for significant gender differences were small to medium (Cohen’s d range: 0.156–0.337), consistent with the broader literature on gender differences in suicide-related variables. Such distinctions highlight the necessity of gender-sensitive preventive and intervention approaches. The observed age-related decline in suicide risk and suicidal ideation, despite increased fearlessness with advancing age, stands in contrast with general populational trends (e.g. 41, 42). Age group differences were statistically significant for most suicide-related outcomes, though effect sizes were small (η² range:.018–.065), indicating that age accounts for a modest proportion of variance. A decline in suicide related variables with age could be explained by the characteristic of the group (i.e. internal factors)- the study only included active veterinary practitioners, i.e. those that have been able to maintain work for many years, not accounting for those that have left the profession. Another explanation refers to a possibly lighter workload or partial retirement in this age group that would contribute to less work-related stress or exposure (external factors).

Although “suicide contagion” was mentioned as a risk factor in previous literature (12), our results did not support this hypothesis. Suicide contagion is defined as direct or indirect exposure to suicide, which can shape attitudes and heighten vulnerability to suicidal behaviors (43). There were no statistically significant links between knowing a colleague who died by suicide, the number of such individuals, or the closeness of the relationship, and measures of suicidal behavior or attitudes toward suicide.

In an article by Bernard E. Rollin (13) the author discusses euthanasia, moral stress, and chronic illness in veterinary medicine, emphasizing the complex ethical dilemmas veterinarians face due to their professional responsibilities and personal values. Moral stress in the context of veterinary practitioners can be defined as stress resulting from the conflict between veterinarians’ original motivation (alleviating animal suffering) and their actual tasks (often performing euthanasia) (44). Moral stress was more relevant in the older age groups in the group comparisons and the network analysis also revealed a connection with Age — possibly due to an accumulation of moral stress with increased or additive work experience. We interpret this pattern as suggesting that moral stress represents a contextual and ethical stressor meaningfully associated with personal and occupational variables (particularly age), but one that does not directly predict core suicide risk indicators in regression models when competing psychological predictors are present. This supports its interpretation as a unique form of distress — moral rather than purely psychological or behavioral — and highlights its potential indirect role in shaping mental health outcomes in veterinary professionals. Drawing on the conceptual framework of Buchbinder et al. (2024) (20), who distinguish moral stress (chronic, systemic) from moral distress (situational, involving powerlessness) and moral injury (lasting damage from transgressive events), the moral stress captured by item SM in this study most closely resembles the systemic, accumulative form of moral burden, particularly given its association with age and professional experience rather than acute events.

With the caveat that E3 is a single, unvalidated item and findings should be interpreted accordingly, the strong predictive link between thoughts of using euthanasia drugs on themselves and both suicidal behavior, ideation and communication as well as the acquired capability for suicide. This finding is important for two reasons: it underscores the role of suicidal ideation in suicidal behavior and capability as well as the crucial aspect of access to lethal means. Access to lethal means has been established to be a practical component of capability for suicide (7) and previous research raised the role access to euthanasia drugs plays in suicide risk among veterinary professionals (12, 45).

Considering the use of euthanasia drugs on yourself emerged as the dominant predictor for the Capability for suicide, far outweighing all other variables. This reinforces the conceptual link between suicidal rehearsal and occupational access to euthanasia methods. Thus, our network analysis further supports this interpretation by identifying self-reported consideration of using euthanasia drugs on oneself as a structural bridge between behavioral and dispositional suicide indicators, suggesting its specificity to suicide-related cognition rather than general psychological distress. This reinforces previous literature highlighting the role of occupational familiarity and access to lethal means in shaping suicide capability (11).

The high prevalence (approximately 24%) of respondents reporting thoughts about using euthanasia drugs for self-harm further underscores the urgent need for targeted interventions. This finding suggests that restricting physical access or implementing rigorous control measures could be a critical component in suicide prevention strategies. A key public health strategy, with strong empirical support, for reducing suicides is limiting access to common means of suicide—an approach known as means restriction (46). Its effectiveness is partly due to the fact that many individuals at highest risk are not reachable through direct intervention and often avoid detection. Suicide attempts tend to be method-specific, so when a preferred method is unavailable, people are less likely to act (46) or may choose a less lethal alternative. From a prevention standpoint, the use of less lethal methods increases the chance of survival in the event of an attempt. Other studies, that investigated medication storage practices (47, 48), although neither directly assessed the impact of formal policies, both found that the use of secure storage methods—such as locked cabinets—was linked to a reduced frequency of reported suicide attempts.

In an article by Bryan et al. (49) (49) the authors discuss the temporal dynamics of suicide risk in an attempt to answer the question on when suicide occurs rather than why. Based on the Fluid Vulnerability Theory (FVT) (50) three types of change processes leading to a shift from low to high suicide probability state have been determined. Stable change implies, mild and smooth fluctuations that will likely return to equilibrium (constituting low risk of progression from suicidal ideation to action). Dysregulated change that encompasses rapid, large fluctuations indicating vulnerability for high risk states and lastly — discontinuous change: Sudden shifts without warning, often described as “out of the blue”. The last type of change, supported by previous research indicating that a considerable amount of individuals who progress to make a suicide attempt make the final decision directly before the attempt and without previous planning (49), is especially important to our findings. In this context occupational access to and extensive knowledge of lethal means — specifically euthanasia drugs — may partially account for the elevated suicide rates observed in veterinary professionals, though causal inference is not possible from this cross-sectional design. As previously mentioned, research indicates, that when deaths due to pentobarbital poisoning are excluded from statistical analyses, the suicide rate among veterinarians is no longer significantly elevated compared to the general population (22). In line with these findings, we postulate that ready access to euthanasia drugs not only facilitates the progression of non-specific suicidal ideation to specific planning (i.e. ideation involving a specific accessible method) but may reduce the typical protective barriers that could otherwise delay or interrupt action during high-risk moments — specifically, veterinarians may be disadvantaged by the reduction of time and logistical barriers that might otherwise create opportunities for intervention or reconsideration during periods of acute risk.

Although there is no empirical possibility to establish whether the intensity of suicidality at the decisive moment is in any way different in veterinarians than in the at-risk portion of the general population, there is also no reason why it would be. Thus, we hypothesize that there may be no difference in specific suicidality distinguishing veterinarians from the general population that increases their mortality rate due to suicide, other than the fact that they are disadvantaged by the absence of the logistical and temporal barriers that typically afford protective moments during periods of acute risk. This hypothesis aligns with Klonsky et al. (4), who argued the elevated suicide risk in anesthesiologists and other medical professionals could be explained by their heightened practical capability regarding lethal means, effectively elevating their acquired capability for suicide to substantially elevated levels. The predominantly private structure of veterinary services in Poland, with limited institutional support, chronic on-call burdens, and absence of centralized mental health infrastructure for veterinary professionals, may amplify the risk factors identified in this study. Prevention strategies must therefore be considered at both the individual and systemic level, accounting for the structural features of veterinary practice in Poland.

The study’s analytical approach, combining traditional statistical methods with machine learning and network analysis, provided nuanced insights into how different psychological, occupational, and social variables interact to influence suicidality. The network analysis revealed that suicide-related outcomes and psychosocial distress form a tightly interconnected web, with suicidal ideation related to euthanasia drugs acting as a crucial bridging element. Taken together, these results reinforce the need to distinguish between situational distress (e.g. occupational), clinical symptomatology (depression, anxiety), moral stress — which, while not a direct predictor of suicidality in regression models, emerged as meaningfully associated with age and occupational experience in network analyses, suggesting a potential indirect pathway — and structural enablers (access to lethal means) in the understanding of suicidal risk factors as well as comprehensive suicide prevention strategies. Our findings build upon and extend previous work by demonstrating the interaction of these factors and informing research on suicides in particular on the ideation to behavior gap.

## 5 Conclusions

This study provides compelling evidence that veterinarians face an elevated risk of suicide not solely due to psychological distress but also because of profession-specific factors—most notably occupational access to lethal means. Our findings confirm that suicidal ideation and behaviors are highly prevalent within this population, with nearly half the respondents scoring above the clinical threshold for suicide risk and nearly one-quarter reporting thoughts of using euthanasia drugs on themselves.

Critically, suicidal ideation involving euthanasia drugs emerged as the most powerful and consistent predictor of both suicidal behavior and acquired capability for suicide. This reinforces the ideation-to-action framework and supports the hypothesis that practical access to lethal means can serve as a key enabler in the transition from suicidal thoughts to actions.

These findings call for urgent, systemic interventions in veterinary practice. Suicide prevention efforts must go beyond individual-level support and include structural strategies such as tighter regulation of euthanasia drug access as well as institutionalized mental health services. Additionally, routine monitoring and early identification of high-risk individuals—especially those expressing specific method-based ideation—should be integrated into veterinary professional standards and practice environments.

By integrating traditional statistical methods with machine learning and network analysis, this study adds depth to our understanding of how occupational, psychological, and environmental factors interact to elevate suicide risk. The results underscore the need for targeted, data-informed suicide prevention approaches tailored to the unique risks faced by veterinary professionals.

## 6 Limitations

Several limitations should be noted. First, the cross-sectional nature of the study precludes any conclusions about causality or temporal relationships between variables. Second, although item E3 proved highly predictive, it is a single-item self-report question and may be influenced by response biases or contextual interpretation. Despite its strong predictive performance in this study, findings based on this item should be interpreted cautiously, as single-item measures are susceptible to response bias, contextual misinterpretation, and ceiling/floor effects. Future research should develop and validate a multi-item instrument for capturing euthanasia-specific suicidal ideation in veterinary populations. Third, while the sample was nationally representative in size and diversity, participation was voluntary and may reflect self-selection by individuals more concerned with or affected by mental health issues. Fourth, moral stress was assessed via a single item (SM) rather than a validated scale. This limits the precision and comparability of moral stress findings with the broader moral injury literature; future research should employ a validated moral injury or moral distress instrument Fifth, the SAW (Support at Work) index demonstrated sub-optimal internal consistency (α=0.559), and the ACWRSS_PFS subscale also fell below the conventional threshold (α=0.646). Findings involving these measures should be interpreted with appropriate caution. Sixth, the survey comprised a substantial number of items; questionnaire fatigue may have affected response quality, particularly for items appearing later in the survey. Eighth, several constructs in the survey overlap conceptually (e.g., burnout, psychological distress, anxiety), which increases the risk of scale contamination. A limitation of our study is the absence of demographic data on racial and ethnic identification as well as cultural or geographic background. These data were not collected because the survey specifically targeted Polish veterinarians, who are largely homogeneous in terms of ethnic background, and the survey was conducted exclusively in Polish and distributed solely via Polish-speaking professional channels. It may be assumed the respondents are predominantly from a Polish-Caucasian background. Future research may benefit from broader demographic sampling to enhance generalizability. Lastly, despite sophisticated analytical techniques, the possibility of omitted variable bias remains. Other potentially influential factors, including personality traits, coping styles, and broader systemic issues within the veterinary profession, might further explain variance in suicide risk and warrant investigation in future studies.

## Data Availability

The raw data supporting the conclusions of this article will be made available by the authors, without undue reservation.
